# The Untapped Potential of Ascon Hash Functions: Benchmarking, Hardware Profiling, and Application Insights for Secure IoT and Blockchain Systems

**DOI:** 10.3390/s25195936

**Published:** 2025-09-23

**Authors:** Meera Gladis Kurian, Yuhua Chen

**Affiliations:** Department of Electrical and Computer Engineering, University of Houston, Houston, TX 77204, USA

**Keywords:** Ascon-Hash256, Ascon-XOF, benchmarking, IoT security, blockchain-enabled IoT, post-quantum cryptography

## Abstract

Hash functions are fundamental components in both cryptographic and non-cryptographic systems, supporting secure authentication, data integrity, fingerprinting, and indexing. While the Ascon family, selected by the National Institute of Standards and Technology (NIST) in 2023 for lightweight cryptography, has been extensively evaluated in its authenticated encryption mode, its hashing and extendable-output variants, namely Ascon-Hash256, Ascon-XOF128, and Ascon-CXOF128, have not received the same level of empirical attention. This paper presents a structured benchmarking study of these hash variants using both the SMHasher framework and custom Python-based simulation environments. SMHasher is used to evaluate statistical and structural robustness under constrained, patterned, and low-entropy input conditions, while Python-based experiments assess application-specific performance in Bloom filter-based replay detection at the network edge, Merkle tree aggregation for blockchain transaction integrity, lightweight device fingerprinting for IoT identity management, and tamper-evident logging for distributed ledgers. We compare the performance of Ascon hashes with widely used cryptographic functions such as SHA3 and BLAKE2s, as well as high-speed non-cryptographic hashes including MurmurHash3 and xxHash. We assess avalanche behavior, diffusion consistency, output bias, and keyset sensitivity while also examining Ascon-XOF’s variable-length output capabilities relative to SHAKE for applications such as domain-separated hashing and lightweight key derivation. Experimental results indicate that Ascon hash functions offer strong diffusion, low statistical bias, and competitive performance across both cryptographic and application-specific domains. These properties make them well suited for deployment in resource-constrained systems, including Internet of Things (IoT) devices, blockchain indexing frameworks, and probabilistic authentication architectures. This study provides the first comprehensive empirical evaluation of Ascon hashing modes and offers new insights into their potential as lightweight, structurally resilient alternatives to established hash functions.

## 1. Introduction

Hash functions are foundational tools in modern computing systems. They enable a wide range of functionalities across both security-critical and performance-oriented domains, including data integrity verification, message authentication, digital signatures, deduplication, and secure indexing [1,2]. In cryptographic applications, key properties such as collision resistance, preimage resistance, and diffusion play a central role in maintaining the integrity and confidentiality of data. Meanwhile, in non-cryptographic contexts, such as hash tables, Bloom filters, fingerprinting systems, and memory-efficient data structures, metrics like uniform distribution, speed, and lightweight implementation are prioritized [3,4,5]. As computing environments evolve to span low-power devices, embedded systems, and resource-constrained Internet of Things (IoT) networks, there is a growing demand for hash functions that can simultaneously offer strong security guarantees and efficient implementation across diverse platforms.

The Ascon family of lightweight cryptographic algorithms, standardized by the National Institute of Standards and Technology (NIST) in 2023 [6], offers a promising set of hashing and authenticated encryption primitives optimized for constrained devices. Among its hash variants, Ascon-Hash256, Ascon-XOF128, and Ascon-CXOF128 adopt a sponge-based structure with a compact, hardware-friendly design, making them attractive for applications that demand both structural integrity and lightweight implementation.

Despite their inclusion in the final NIST Lightweight Cryptography (LWC) standard portfolio, empirical benchmarking of Ascon hash variants remains limited. While Ascon-AEAD128, the Authenticated Encryption with Associated Data (AEAD) variant, has been extensively evaluated in software and hardware implementations across 8-bit, 32-bit, and 64-bit microcontrollers, as well as in Field-Programmable Gate Array (FPGA) and Application-Specific Integrated Circuit (ASIC) platforms [7,8], the hash variants have not received similar benchmarking attention. Public benchmarking platforms such as SUPERCOP and eBACS do not currently include these variants by default [9,10]. Furthermore, there is a notable lack of published comparisons involving Ascon hashes and other widely deployed cryptographic hash functions such as Secure Hash Algorithm 3 (SHA3) [11], SHAKE256 [12], and BLAKE2s [13]. Even fewer studies have explored their performance in structural or low-entropy input domains, where non-cryptographic hashes like MurmurHash3 [14] and xxHash [15] are commonly used.

Although formal security proofs and design rationales for Ascon hash functions are available through the NIST standardization process [6], there remains a gap in our understanding of their empirical behavior under real-world input patterns. Structural properties such as avalanche diffusion, output bias, collision distribution under sparse or patterned inputs, and variable-length adaptability are increasingly relevant in emerging applications such as packet fingerprinting [3], Bloom filter-based replay detection [16], blockchain transaction indexing [17], and lightweight key derivation in embedded systems [18]. For example, Ascon-XOF’s support for variable-length output makes it directly comparable to the SHAKE family of extendable-output functions in applications involving domain-separated hashing, key derivation, and extendable identifiers within post-quantum secure protocols.

This paper addresses the above gaps by presenting the first structured empirical benchmarking of the Ascon hash variants, with a focus on their statistical and structural behavior under practical input conditions. We use the SMHasher test suite to evaluate properties such as avalanche bias, permutation sensitivity, and keyset diffusion. While originally developed for non-cryptographic hash functions [19], SMHasher remains a valuable framework for quantifying bit-mixing quality, output uniformity, and resistance to structural bias under diverse inputs. We emphasize that SMHasher is not a cryptanalytic tool and does not replace formal security evaluation; rather, its tests complement traditional analysis by exposing structural weaknesses that may impact performance or correctness in practical applications. It is important to note that the cryptographic soundness of Ascon has already been extensively vetted through the Competition for Authenticated Encryption: Security, Applicability, and Robustness (CAESAR) and the NIST Lightweight Cryptography process, where it was selected as the final standard. Our contribution therefore complements this foundation by focusing on benchmarking, hardware profiling, and application-level evaluations in IoT and blockchain contexts. These metrics are particularly relevant in constrained scenarios such as replay detection and Bloom filter-based lookups, where statistical uniformity and diffusion directly influence system behavior.

In addition to statistical analysis, we evaluate the applicability of Ascon hash variants in broader domains, including secure indexing frameworks, post-quantum digital signatures, IoT fingerprinting, and blockchain-based integrity verification. These domains are highly sensitive to properties such as the collision behavior, bit-level diffusion, and output distribution bias, which directly affect system performance and correctness. Our benchmarking compares Ascon-Hash256 against established cryptographic hashes such as SHA3-256 and BLAKE2s, as well as widely used non-cryptographic alternatives including MurmurHash3 and xxHash. This comparative evaluation highlights the untapped versatility of the Ascon family beyond its AEAD origins, demonstrating its suitability for both security-critical protocols and resource-aware structural operations. Our results further underscore the unique combination of efficiency, diffusion, and structural robustness that Ascon hashes provide.

In summary, this paper makes the following key contributions:

1. It provides the first comprehensive empirical benchmarking of Ascon-Hash using SMHasher, uncovering structural properties such as avalanche bias, diffusion, and robustness under low-entropy inputs.
2. It offers a comparative evaluation of Ascon hash variants against cryptographic standards (SHA3-256, SHAKE256, BLAKE2s) and high-speed non-cryptographic hashes (MurmurHash3, XXHash), highlighting their relative efficiency and statistical behavior.
3. It demonstrates the practical applicability of Ascon hashes in lightweight contexts, including Bloom filter replay detection, Merkle tree aggregation, device fingerprinting, and tamper-evident logging for IoT and blockchain systems.

These findings underscore the untapped potential of Ascon hash functions and provide new insights into their practical relevance across domains that require a balance of cryptographic strength, lightweight design, and structural integrity [3,20,21]. The remainder of this paper is organized as follows: Section 2 reviews related work, and an overview of the Ascon hashing algorithm is given in Section 3. Section 4 details the methodology, including the benchmarking environment, comparison scope, and both SMHasher and Python-based evaluation frameworks. Section 5 presents results across structural benchmarking, replay prevention, post-quantum integration, fingerprinting applications, and Merkle tree diffusion. Section 6 discusses the broader implications of these findings, and Section 7 concludes the paper.

## 2. Related Work

Hash functions are essential components of modern cryptographic systems, providing compact representations of data that are essential for integrity verification, authentication, and digital signatures [22,23]. A hash function takes an input of arbitrary length and maps it to a fixed-size bit string known as the message digest or fingerprint. Formally, a hash function *h* is defined as follows:

$$h:{\left\{0,1\right\}}^{*}\to {\left\{0,1\right\}}^{n}$$

where *n*, the output length of the hash, is typically between 256 and 512 bits. Unlike encryption schemes, hash functions are keyless and non-reversible, making them ideal for applications where verification rather than confidentiality is required [24].

To be cryptographically secure, hash functions must satisfy three main properties [1]: Preimage resistance ensures that it is computationally infeasible to recover the original message given only its hash. Second preimage resistance means that it is computationally infeasible to find a different input with the same hash as a given message. Lastly, collision resistance makes it infeasible to find any two distinct messages that produce the same hash output. These properties underpin many cryptographic protocols, including digital signatures and message authentication codes [25].

Early dedicated hash functions such as MD4 [26] and MD5 [27], designed by Ronald Rivest, were optimized for software efficiency using 32-bit word operations and Boolean logic. Although widely adopted, both functions eventually proved vulnerable to collision attacks [28]. For instance, collisions in MD5 were discovered in 2004, undermining its use in critical security protocols. To address these weaknesses, the NIST introduced the Secure Hash Algorithm (SHA) family, beginning with SHA-0 in 1993, followed by SHA-1 in 1995. However, SHA-1 was eventually broken too, with a collision discovered in $2^{63}$ steps [29].

This led to the development of the SHA-2 family, comprising SHA-224, SHA-256,  SHA-384, and SHA-512, which remain widely used and are currently considered secure [30]. Seeking further diversification, the NIST launched a public competition in 2007 to design a new hash function with a different internal structure [31]. The winner, Keccak, became the basis for SHA-3, which was standardized in 2015 [11]. Unlike earlier Merkle–Damgård constructions, SHA-3 relies on the sponge construction [32], which consists of an absorbing phase (input processing) and a squeezing phase (output generation). This flexible architecture allows the creation of both traditional hash functions and Xtendable Output Functions (XOFs), such as SHAKE128 and SHAKE256 [11].

In addition to dedicated designs, hash functions can also be constructed from block cipher primitives. The Matyas–Meyer–Oseas (MMO) construction, for example, derives a compression function from an existing block cipher [22]. Since the Advanced Encryption Standard (AES) became a global standard with widespread hardware acceleration [33,34], numerous proposals have leveraged the AES to build hash functions that exploit this speed and availability [35]. These approaches avoid the need to implement a new primitive in the hardware and instead reuse the AES, thereby reducing both area and power consumption in constrained devices.

With the rise of the IoT, there has been growing interest in lightweight hash functions that balance security with efficiency on resource-constrained platforms [36,37]. In August 2025, the NIST LWC project finalized the Ascon family as the standard for protecting such devices [6]. While Ascon is primarily recommended for authenticated encryption with associated data, the family also includes Ascon-Hash256, Ascon-XOF128, and Ascon-CXOF128, which inherit the sponge-based structure of Keccak but are optimized for lightweight use [38]. These functions provide excellent avalanche characteristics, uniform output distribution, and low implementation costs in terms of hardware, making them promising candidates for IoT authentication, probabilistic data structures such as Bloom filters [16], and Merkle tree-based applications.

Quantum computing presents a significant challenge to existing cryptographic schemes. Peter Shor’s breakthrough algorithms in the mid-1990s showed that Rivest–Shamir–Adleman (RSA) and elliptic-curve cryptosystems could be broken in polynomial time on quantum computers [39]. Shor’s period-finding and discrete logarithm algorithms render all widely used public-key systems insecure in the quantum setting. Additionally, Grover’s algorithm reduces the brute-force complexity of symmetric-key operations from $2^{n}$ to $2^{n/2}$, necessitating longer key and hash lengths for adequate protection [40].

As a result, the field of post-quantum cryptography (PQC) has expanded to include alternatives resilient to quantum attacks [41]. These include lattice-based (e.g., Kyber, Dilithium), code-based (e.g., McEliece), and hash-based schemes [1]. Hash-based digital signatures, such as SPHINCS+, rely solely on the properties of cryptographic hash functions and are regarded as strong candidates for long-term post-quantum security [42].

**Figure 1:**
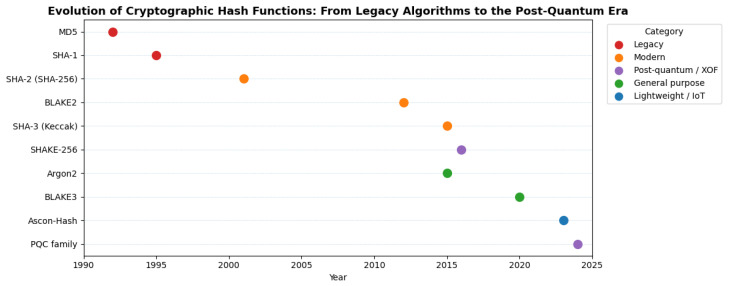
Timeline illustrating the evolution of cryptographic hash functions from legacy algorithms (e.g., MD5, SHA-1) through modern constructions (e.g., SHA-2, SHA-3, BLAKE2/3, Ascon-Hash) to the emerging post-quantum cryptography(PQC) era. Categories are distinguished by color as follows: legacy/insecure (deprecated designs no longer recommended for security use), modern secure (currently standardized and widely deployed), lightweight (optimized for constrained devices and IoT), and PQC-ready (designed or adapted for quantum-resistant applications). This categorization highlights each function’s security role, application domain, and relevance in current cryptographic standards.

Figure 1 provides a visual timeline of cryptographic hash function adoption. It highlights the trajectory from early designs (e.g., MD5, SHA-1), which are now deprecated, to more modern, domain-optimized alternatives like SHAKE256, BLAKE3, and Ascon-Hash. The recent standardization of Ascon by the NIST marks a shift toward lightweight and structurally secure hash functions, motivating the need for empirical benchmarking as presented in this work.

The versatile nature of hash functions has led to their widespread use in digital signatures, message authentication codes, password hashing, blockchain transaction validation, pseudorandom number generation, and replay detection mechanisms [2,16,20]. In many of these domains, especially in constrained or adversarial settings such as edge computing and decentralized systems, the demand for efficient, secure, and adaptable hash functions is growing [43,44]. Lightweight hashes are also increasingly explored in post-quantum signature schemes and blockchain-based IoT authentication frameworks, further underscoring the need for domain-specific benchmarking [20,45,46,47,48].

While prior work has explored both classical and quantum-safe hash designs [49], there has been limited empirical evaluation of the domain-specific benefits of sponge-based lightweight hash functions such as Ascon. This work addresses that gap by benchmarking Ascon-Hash in cryptographic and structural applications, examining its potential to replace or complement SHA3, BLAKE2s, and non-cryptographic hashes in secure indexing, fingerprinting, and blockchain-based aggregation.

## 3. Overview of the Ascon Hashing Algorithm

The Ascon hashing functions are derived from the Ascon family of lightweight cryptographic primitives, selected as the primary standard in the NIST-LWC competition in 2023 [6]. While originally proposed for AEAD, the design was later extended to include sponge-based hash functions: Ascon-Hash256, Ascon-XOF128, and Ascon-CXOF128. These variants aim to combine strong cryptographic properties with low implementation costs, making them ideal for applications in embedded systems, secure indexing, and constrained IoT environments.

All Ascon hash variants are built upon a sponge construction that processes data in two phases: the absorbing phase, where input blocks are XORed into the internal state, and the squeezing phase, which produces the output hash digest. The internal state of all Ascon’s hashing variants are 320 bits, divided into a 64-bit rate (r = 64) and a 256-bit capacity (c = 256). The number of permutation rounds applied per block is 12 for all three variants to maximize diffusion.

**Figure 2:**
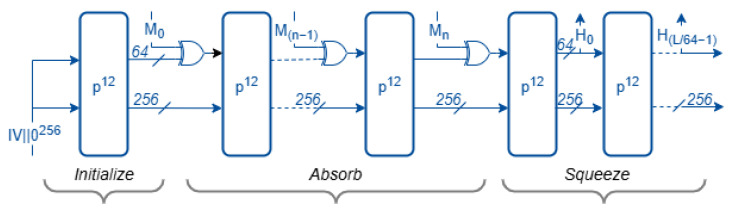
Hashing mode for Ascon-Hash256 and Ascon-XOF128.

The mode of operation for Ascon-Hash256 and Ascon-XOF128, illustrated in Figure 2, consists of three primary phases: initialization, message absorption, and output squeezing. The construction takes a variable-length message *M* as input. For Ascon-Hash256, the output length *L* is fixed at 256 bits, whereas, in Ascon-XOF128, the output length is variable. The initialization vector (IV) for Ascon-Hash256 is 0x0000080100cc0002, while that for Ascon-XOF128 is 0x0000080000cc0003. In Ascon-XOF128 and Ascon-CXOF128, the suffix “128” denotes the intended security strength rather than the output size.

**Figure 3:**
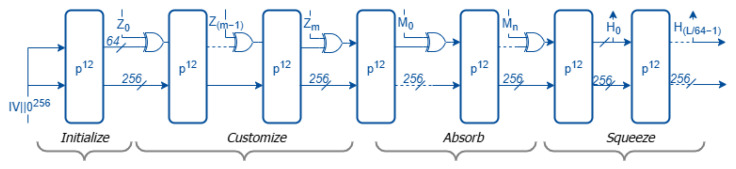
Hashing mode for Ascon-CXOF128.

The customized variant of Ascon-XOF128, referred to as Ascon-CXOF128, is shown in Figure 3 and extends the base functionality by allowing the inclusion of a customization string *Z* in the computation. For the same input message, two customized XOF instances using different customization strings will yield distinct outputs.

Ascon-CXOF128 differs from Ascon-XOF128 in the following aspects [6]:

- Domain separation: Ascon-CXOF128 uses a different initialization vector (IV) from Ascon-XOF128 and supports user-defined customization strings. The IV for Ascon-CXOF128 is 0x0000080000cc0004. For instance, Ascon-CXOF128 enables domain separation by allowing parameters such as output lengths or application-specific identifiers to be encoded into the customization string. This guarantees that outputs derived in different contexts (e.g., key derivation, Merkle tree hashing, or protocol identifiers) remain distinct, even if the same input message is used.
- Additional input: Alongside the message, Ascon-CXOF128 accepts a customization string *Z* whose length is at most 2048 bits (256 bytes).
- Input formatting: The customization string *Z* is prepended to the message blocks as follows:
  $$Z_{0}\;∥\;Z_{1}\;∥\;\ldots \;∥\;Z_{m}\;∥\;M_{0}\;∥\;\ldots \;∥\;M_{n-1}\;∥\;M_{n},$$
  where $Z_{0}$ is a 64-bit integer denoting the bit length of the customization string, and $Z_{1},\ldots ,Z_{m}$ are 64-bit blocks obtained by parsing and padding *Z*.

### Ascon Permutation

In Ascon’s sponge construction, the core primitive is a permutation on a 320-bit internal state:

(1) $$S=S_{0}\;∥\;S_{1}\;∥\;S_{2}\;∥\;S_{3}\;∥\;S_{4}$$

where each $S_{i}$ is a 64-bit word (0≤i≤4). Each round consists of three sequential layers: constant addition ($p_{c}$), substitution ($p_{S}$), and linear diffusion ($p_{L}$).

**Figure 4:**
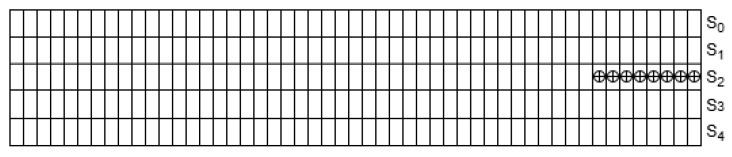
Constant addition layer ($p_{c}$) where a round constant $const_{i}$ is XORed into one state word [6].

(i) Constant Addition Layer ($p_{c}$): A round-dependent constant $const_{i}$, listed in Table 1, is XORed into the least significant bits of one state word to break the symmetry between rounds and prevent slide attacks, ensuring distinct evolution of the state across different rounds. The standard specifies round constants for up to 16 rounds to accommodate potential functionality extensions in the future [6]. Figure 4 illustrates this process.

(ii) Substitution Layer ($p_{S}$): This layer applies a single 5-bit S-box in parallel to each of the 64-bit slices of the 5×64-bit state:

$$\left(s_{0,j},s_{1,j},\ldots ,s_{4,j}\right)\to SBox\left(s_{0,j},s_{1,j},\ldots ,s_{4,j}\right),\;0\leq j<64.$$

**Figure 5:**
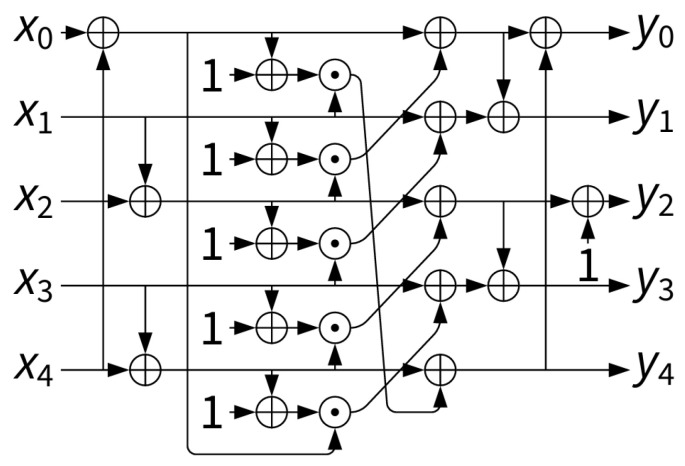
The 5-bit S-box used in the Ascon permutation [50].

**Figure 6:**
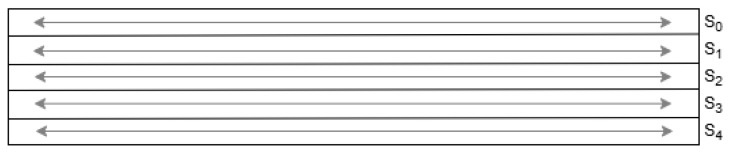
Linear diffusion layer ($p_{L}$) where each word is XORed with two rotated versions of itself [6].

It is hardware efficient, requiring only XOR, AND, and NOT operations, yet provides strong nonlinearity. A circuit representation of the S-box is shown in Figure 5, while its lookup table mapping is given in Table 2.

(iii) Linear Diffusion Layer ($p_{L}$): This layer improves avalanche properties by XORing each word with rotated versions of itself, using constants specific to each word index. The transformations are illustrated in Figure 6 and defined in Equations (2)–(6):

(2) $$\Sigma_{0}\left(S_{0}\right)=S_{0}⊕\left(S_{0}⋙19\right)⊕\left(S_{0}⋙28\right)$$

(3) $$\Sigma_{1}\left(S_{1}\right)=S_{1}⊕\left(S_{1}⋙61\right)⊕\left(S_{1}⋙39\right)$$

(4) $$\Sigma_{2}\left(S_{2}\right)=S_{2}⊕\left(S_{2}⋙1\right)⊕\left(S_{2}⋙6\right)$$

(5) $$\Sigma_{3}\left(S_{3}\right)=S_{3}⊕\left(S_{3}⋙10\right)⊕\left(S_{3}⋙17\right)$$

(6) $$\Sigma_{4}\left(S_{4}\right)=S_{4}⊕\left(S_{4}⋙7\right)⊕\left(S_{4}⋙41\right)$$

where ⋙ denotes rotation to the right.

Each layer is constant-time, to mitigate timing-based side channel attacks. The combination of symmetry-breaking constants, strong nonlinearity, and rapid diffusion provides robust security for constrained and adversarial environments [50].

The functional characteristics of each hash variant are summarized in Table 3. Ascon-Hash is designed for fixed-length output (256 bits) and is ideal for message authentication, fingerprinting, and digital signature schemes. Ascon-XOF supports variable-length output and is therefore suitable for applications such as key derivation, hierarchical hashing, and domain-separated protocol identifiers. Ascon-CXOF further introduces a context string during initialization to provide built-in domain separation, enabling cryptographic agility across protocols or device layers.

From an implementation standpoint, Ascon hashing functions are optimized for compactness and performance. The sponge-based structure combined with the lightweight permutation core enables very small hardware footprints in ASIC designs [51] and efficient implementations on microcontrollers with limited instruction sets [52]. The algorithm uses only bitwise operations (AND, XOR, NOT) and shift/rotation logic, which are naturally suited to embedded systems and FPGA platforms.

Security-wise, Ascon hash variants maintain strong bounds on preimage and collision resistance. The 256-bit digest in Ascon-Hash provides a classical security margin of $2^{128}$ against collisions. All variants employ a sponge capacity of 256 bits, ensuring that Grover-style quantum attacks cannot reduce preimage resistance below $2^{128}$ [40]. At the implementation level, all operations are constant-time and avoid data-dependent branching, thereby mitigating timing-based side channel attacks. Resistance against more advanced side channels (e.g., power or EM analysis) has been studied separately [52,53,54] and can be enhanced through masking and other countermeasures. Together, these characteristics make Ascon hash functions attractive not only for classical cryptographic applications but also for emerging domains that demand low-power, side channel-resistant, and structurally sound hashing primitives.

## 4. Methodology

This study aims to evaluate the statistical robustness, structural diffusion, and practical applicability of the Ascon hash family in comparison with both cryptographic and non-cryptographic alternatives. To achieve this, we adopted a twofold methodology combining empirical benchmarking and targeted simulations. First, we used the SMHasher framework to provide a standardized and reproducible evaluation of avalanche behavior, output bias, and resistance to structured or low-entropy inputs. These tests were applied uniformly across a set of five representative hash functions to ensure fair comparison. Second, we complemented the SMHasher results with custom Python-based simulations designed to explore application-driven scenarios such as Bloom filter indexing, log fingerprinting, and Merkle tree diffusion. This dual approach allowed us to capture both the baseline statistical properties of the candidate hash functions and their performance in practical, domain-relevant contexts.

### 4.1. Benchmarking Framework and Environment

All evaluations were conducted using the SMHasher framework [19], a widely used benchmarking suite designed to assess the statistical properties, structural robustness, and runtime behavior of hash functions. While originally designed for non-cryptographic hash functions, SMHasher includes tests that are equally informative for lightweight and cryptographic hash functions, especially in scenarios involving structured, adversarial, or low-entropy inputs.

Benchmarks were performed on a Linux workstation equipped with an Intel Core i7-1165G7 CPU running at 2.91 GHz. The SMHasher suite was compiled using g++ with the -O3 optimization flag to ensure consistent high-performance execution. All tests were conducted under Ubuntu 22.04 LTS (64 bit). All hash outputs were standardized to 256 bits to ensure fair comparisons.

### 4.2. Hash Function Selection and Comparison Scope

This study evaluates five hash functions, including both cryptographic and non-cryptographic types. The cryptographic group consists of Ascon-Hash256, SHA3-256, and BLAKE2s. These hash functions were empirically benchmarked using the SMHasher framework, as supported by the actively maintained repository by rurban [19]. SHA3-256 and BLAKE2s were selected for their standardized status and relevance to post-quantum and lightweight cryptographic applications. While SHAKE256 and Ascon-XOF are not natively supported in SMHasher, they are included in the comparative conceptual analysis due to their sponge-based structure and functional similarity to SHA3-256 and Ascon-Hash256, respectively.

The non-cryptographic group comprises MurmurHash3 and xxHash, both widely adopted in performance-critical domains such as hash tables, Bloom filters, and software indexing. These were included to emphasize statistical and structural differences between cryptographic and high-speed hash functions, particularly in terms of diffusion, bias resistance, and collision behavior under structured inputs.

Although the primary focus of this study is the empirical evaluation of Ascon-Hash256 using SMHasher, a complementary specification-based analysis of Ascon-XOF128 and SHAKE256 is also included, based on their official specifications and the prior literature. Since all Ascon hash variants share the same sponge structure and permutation, only differing in rate, output format, and optional customization, Ascon-CXOF128 is not separately analyzed here. Because SMHasher does not support variable-length output functions, runtime metrics for SHAKE256 and Ascon-XOF could not be collected directly. Instead, their architectural features, extensibility, and suitability for applications such as domain-separated hashing, key derivation, and hierarchical tree hashing are discussed in a dedicated section.

### 4.3. Test Categories in SMHasher

The following SMHasher test categories were used to evaluate the statistical and structural robustness of each hash function:

- Avalanche Test: This test measures the bit diffusion strength of a hash function by evaluating how a single-bit change in the input affects the output [55]. For each input length, thousands of input pairs differing by exactly one bit are hashed. The percentage of output bits that flip is recorded for each case. Ideally, each output bit should flip with 50% probability, indicating perfect avalanche behavior. SMHasher reports the worst-case output bit bias, defined as the maximum deviation from this ideal across all output bits. A low bias indicates strong mixing and good diffusion properties.
- Keyset Tests: SMHasher includes several structured key categories, namely sparse, permutation, and cyclic inputs, that simulate constrained, low-entropy, or patterned input conditions [19]. These tests evaluate how well a hash function maintains uniformity, diffusion, and collision resistance in adversarial or real-world scenarios.
  *Sparse Test:* This test simulates low-entropy input conditions by generating keys with only a few active bits. It models use cases such as feature flags, protocol identifiers, and sparse data encodings. A robust hash function should diffuse these small changes evenly and avoid output bias or clustering.
  *Permutation Test:* Keys are generated by selecting up to seven values from a pool of eight fixed blocks, simulating the structured inputs often seen in memory-constrained systems, cryptographic identifiers, or header formats. This test reveals how the hash function handles repeated structures and limited entropy sources.
  *Cyclic Test:* This test evaluates the hash function’s behavior on periodic and repeating input patterns, such as those found in network packet headers, sensor data streams, or protocol padding. The hash function must maintain randomness and collision resistance, even when input entropy is low or highly regular.
  *Zeroes Test:* This test detects output bias by measuring the frequency of zero bits across all hash outputs. A well-designed hash function should exhibit a near-random distribution of zeros and ones. Excessive zeroes may indicate insufficient diffusion or predictable output bits, especially in the most or least significant positions.
- Bit Distribution and Bias: This test evaluates whether the hash function’s output bits are uniformly distributed across different input conditions. It identifies skewed or biased bits that may reduce the randomness or security of the hash output.

All hash functions were tested under identical system configurations and repeated across sufficient trials to ensure statistical reliability. To enable uniform comparison across functions with different output lengths, all hash outputs were truncated or zero-padded to 256 bits where applicable.

### 4.4. Python Simulation Framework

All custom simulations were implemented in Python 3.10. Ascon-Hash256 and Ascon-XOF were instantiated using the xoflib package, while the Bloom filter was managed with the bloom_filter library. For baseline comparisons, SHA3-256 and BLAKE2s were obtained from the Python standard library hashlib, whereas SHAKE256 and an alternative SHA3-256 implementation were sourced from PyCryptodome (Crypto.Hash). MurmurHash3 was provided by the mmh3 package, and xxHash by the xxhash package. Supporting utilities included itertools, random, and statistics from the Python standard library.

In the Bloom filter experiments (Section 5.2), we instantiated a filter of size $m=10^{5}$ bits with k=10 independent hash indices, following standard Bloom filter notation [56]. Each 128-bit nonce was expanded by Ascon-Hash256 (rate = 64, capacity = 256) using the standard 12-round permutation to derive ten 32-bit pseudorandom indices, with parallel experiments conducted using SHA3-256, Blake2s-256, MurmurHash3, and xxHash for comparison. A total of n=200,000 sequential nonces were inserted, and the False Positive Rate (FPR) was measured after every 10,000 insertions. Results, averaged over five independent runs, were compared against the theoretical Bloom filter model given by Equation (7) [5]:

(7) $$\mathrm{FPR}\left(n,m,k\right)\approx {\left(1-e^{-kn/m}\right)}^{k}$$

which served as a baseline for evaluating how different hash functions affect Bloom filter saturation and FPR stability.

For log fingerprinting [57] (Section 5.4), ten structured log messages (L1–L10) differing by single-field edits (e.g., temperature, timestamp, status) were hashed using Ascon-Hash256, SHA3-256, SHAKE256, BLAKE2s-256, xxHash, and MurmurHash3 with all outputs standardized to 256 bits by truncation or zero-padding for uniform comparison. Avalanche scores for each adjacent log pair were computed as follows:


def bit_diff(h1, h2):
    return sum(bin(a ^ b).count(“1”) for a, b in zip(h1, h2))


The average bit difference across all nine pairs was reported.

For Merkle tree diffusion (Section 5.5), we generated full binary trees up to depth 4 (16 leaves) [58], perturbing each leaf and recording bit diffusion statistics at every level. To evaluate how this behavior scales, we extended the analysis to deeper trees with $2^{4}$, $2^{6}$, $2^{8}$, and $2^{10}$ leaves. In each of 1000 trials per tree size, a single leaf bit was flipped at a random position, and the tree was hashed bottom-up using Ascon-Hash256 and SHA3-256. Using the same bit_diff function, we reported the mean, variance, minimum, and maximum of root-level bit differences.

## 5. Results

### 5.1. Structural Benchmarking Using SMHasher Suite

To assess the suitability of Ascon hash variants for structural applications such as hash tables, Bloom filters, and sensor fingerprinting, we evaluated its statistical robustness using keyset-based tests from the SMHasher suite [19]. These tests simulate adversarial or structured input patterns that are commonly encountered in real-world systems relying on hash-based indexing.

Table 4 consolidates the key results across five dimensions: worst-case avalanche bias, sensitivity to sparse inputs, resilience to structural bias (Permutation and Cyclic Tests), and bit uniformity (Zeroes Test). Compared to other cryptographic hashes (SHA3-256 and BLAKE2s) and widely used non-cryptographic hashes (MurmurHash3 and xxHash), Ascon-Hash256 consistently exhibits stronger or comparable behavior across all metrics.

Ascon-Hash256 achieves the highest score in the Sparse Test (0.968%), indicating strong sensitivity to low-entropy inputs, which is a desirable property for systems that rely on fine-grained feature differentiation, such as Bloom filters or compressed data matching [5]. It also exhibits the lowest structural bias in the Permutation Test (0.081%) and one of the lowest scores in the Cyclic Test (0.151%), demonstrating robust randomness and collision resistance even under highly repetitive input patterns.

In the Zeroes Test, Ascon-Hash256 maintains a balanced output distribution (0.322%), on par with SHA3-256 and significantly more uniform than BLAKE2s. Finally, the worst-case avalanche bias [38] for Ascon-Hash256 remains below 0.83%, validating its strong bit diffusion characteristics across all key sizes. This is superior to SHA3-256 (1.01%) and BLAKE2s (0.85%) and comparable to optimized non-cryptographic hashes, without sacrificing cryptographic integrity.

These results suggest that Ascon-Hash256 offers a compelling alternative to non-cryptographic hashes for indexing and distribution tasks, particularly in applications requiring lightweight implementation, structural robustness, and moderate security guarantees. Its performance reinforces its viability in constrained domains such as embedded systems, sensor networks, and blockchain-based data structures where uniformity and low collision rates are critical [59,60].

Moreover, Ascon-Hash256 exhibits low output bias, strong avalanche characteristics, and uniform output distributions, as validated through SMHasher evaluations conducted over 300,000 input variants, systematically generated by the test suite through single-bit toggling and structured key patterns across varying input lengths. These properties align with the stringent mixing requirements for pseudorandom string derivation (e.g., $R=\mathrm{Hash}({\mathrm{SK}}_{\mathrm{prf}}||\mathrm{OptRand}||M)$) and multi-layer Merkle hashing (e.g., $\mathrm{Digest}=\mathrm{XOF}(R||{\mathrm{PK}}_{\mathrm{seed}}||{\mathrm{PK}}_{\mathrm{root}}||M)$) [49,58], ensuring cryptographic robustness while maintaining implementation efficiency.

### 5.2. Blockchain-Enabled Replay Attack Mitigation for IoT Edge Devices

In blockchain-enabled IoT environments, replay attacks pose a critical threat to both device security and ledger integrity. Adversaries may attempt to reuse valid authentication messages, which can not only grant unauthorized access to IoT nodes but also pollute distributed ledgers with duplicated or fraudulent entries. Such attacks undermine consensus mechanisms, inflate storage requirements, and weaken the trust model that blockchain is designed to provide. To mitigate this risk, we previously proposed a replay protection mechanism for Ascon-AEAD128, integrating a Bloom filter with cryptographically secure hashing using Ascon-XOF128 [16]. In this design, each incoming nonce is processed by the Ascon-XOF128 hashing stage, producing a 256-bit digest that is partitioned into *k* fixed-width indices for bit setting in a Block RAM (BRAM) based Bloom filter. This ensures that duplicate or replayed packets are rejected at the IoT edge before they are forwarded to blockchain storage, thereby preserving ledger consistency while minimizing overhead on resource-constrained devices.

**Figure 7:**
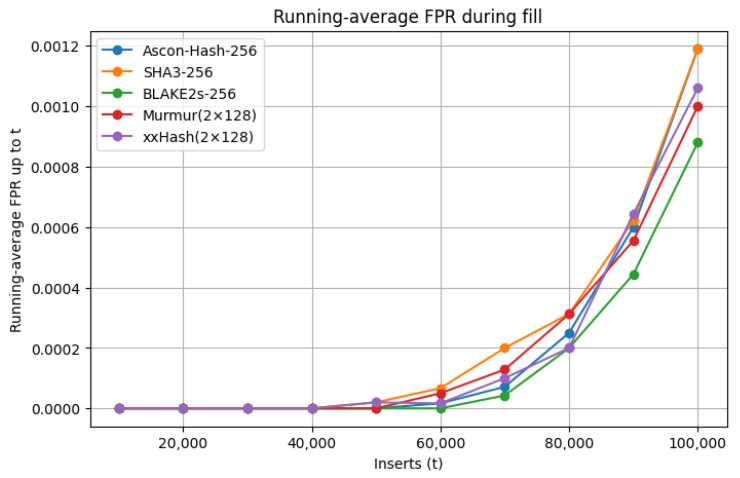
Running-average false-positive rate (FPR) during Bloom filter fill for Ascon-Hash256, SHA3-256, BLAKE2s-256, MurmurHash, and xxHash with $m=2^{20}$, k=10, and $n=10^{5}$ insertions. The Ascon-Hash256 trajectory closely follows that of the fastest non-cryptographic hashes while preserving cryptographic security guarantees. In the context of blockchain-enabled IoT systems, this stability ensures that replayed or duplicate messages are filtered at the edge before being committed to distributed ledgers, thereby reducing ledger pollution and strengthening transaction integrity.

The statistical robustness of Ascon-Hash256, as established in Section 5.1 through SMHasher keyset evaluations, further supports this design choice. Its low avalanche bias (0.823%) and minimal structural bias in Permutation (0.081%) and Cyclic (0.151%) Tests confirm near-uniform index generation even under adversarially structured nonce patterns. This statistical strength is reflected in the Bloom filter’s stable FPR trajectory, shown in Figure 7, which compares Ascon-Hash256 against SHA3-256, BLAKE2s-256, MurmurHash, and xxHash using a filter of size of $m=2^{20}$ bits, k=10 indices, and $n=10^{5}$ inserted elements. All trajectories were obtained through a Python-based simulation, where sequential nonces were inserted and the running FPR was recorded every 1000 insertions and averaged across five trials.

The FPR trajectory for Ascon-Hash256 closely follows that of the fastest non-cryptographic hashes, demonstrating that adopting a cryptographic primitive incurs no measurable penalty in Bloom filter efficiency. The gradual rise in FPR with higher insertion counts reflects the natural saturation behavior predicted by the Bloom filter model, rather than any deficiency in the hash function’s diffusion [56]. In comparison, SHA3-256 exhibits a steeper rise near saturation and BLAKE2s-256 shows minor deviations from the theoretical curve, whereas Ascon-Hash256 remains consistently aligned with the lightweight baselines. This underscores Ascon’s ability to combine Bloom filter efficiency with cryptographic strength, achieving statistical uniformity that surpasses established cryptographic primitives.

While MurmurHash and xxHash achieve comparable FPRs in benign conditions, their SMHasher profiles reveal higher susceptibility to certain structured input patterns, particularly in the Sparse and Cyclic Tests (Table 4). Such weaknesses can be exploited in adversarial environments, where an attacker may deliberately craft nonces to bias bit setting within the Bloom filter, increasing collision probability and degrading replay detection reliability. In contrast, Ascon-Hash256 combines consistently low avalanche bias (0.823%) with the highest Sparse Test score (0.968%) and minimal structural bias in both the Permutation (0.081%) and Cyclic (0.151%) Tests. Together, these properties ensure near-uniform index generation even under hostile input conditions. These statistical guarantees, validated through SMHasher evaluations, directly explain the stable FPR curve observed in Figure 7. Hardware profiling of Ascon hash cores on FPGAs has been reported in prior work [16], confirming their efficiency for lightweight deployment in constrained environments.

Furthermore, Ascon-based hashing inherits the NIST LWC standardized security properties of the Ascon permutation, offering collision resistance, preimage resistance, and robustness against targeted bit-flooding attacks [21]. This combination of statistical uniformity, operational efficiency, and cryptographic strength makes it a compelling choice not only for adversarial IoT edge environments but also for blockchain-enabled IoT systems, where preventing replayed transactions before ledger commitment is critical in maintaining consensus integrity and avoiding ledger pollution [61]. By filtering duplicates and adversarial inputs at the edge, Ascon-based replay prevention reduces computational overhead on blockchain nodes, ensuring scalability and energy efficiency in secure IoT–blockchain deployments.

### 5.3. Lightweight Hash Integration in PQC Signatures and Blockchain-Enabled IoT Authentication

Recent research efforts have explored hybridizing post-quantum digital signatures with lightweight hashing to improve efficiency on resource-constrained platforms. SPHINCS+ was selected as a finalist in the NIST post-quantum cryptography standardization process, reinforcing the importance of optimizing components for constrained platforms. Notably, Magyari et al. [62] proposed a variant of SPHINCS+ known as *Ascon-Sign*, where the default SHAKE256-based hashing components were replaced with Ascon-Hash and Ascon-XOF. Their design retained SPHINCS+’s structural components, including pseudorandom string generation, Forest of Random Subsets (FORS) tree traversal, and eXtended Merkle Signature Scheme (XMSS)-based hyper tree authentication, while substituting the message digest and tree node hashing functions with Ascon primitives.

A comparison of Ascon-Sign against SPHINCS+ and SPHINCS-256 in FPGA deployments is shown in Table 5. Ascon-Sign achieves the lowest resource footprint, requiring only 7.0k Lookup Tables (LUT) and 1 BRAM block on an Artix-7, compared to the 48–51k LUTs and 11.5–22.5 BRAMs needed by SPHINCS+ variants. Although the signing latency of Ascon-Sign is higher (822.3 ms at 100 MHz), its energy consumption ($E_{\mathrm{sign}}$ = 170 mWs) remains competitive for low-duty-cycle IoT applications.

#### Security Bounds and Applicability

The security of post-quantum hash-based signatures is tightly coupled with the diffusion and randomness properties of the underlying hash functions. The cryptanalytic security of Ascon has been extensively vetted during the NIST Lightweight Cryptography process. Table 6 summarizes the theoretical collision, preimage, and second preimage bounds of the Ascon hash variants [6] alongside SHA3 and SHAKE functions [12], which are commonly used in SPHINCS+ and other PQC signature schemes.

The theoretical security bounds of Ascon-Hash256 and Ascon-XOF128 match those of SHA3-256 and SHAKE128 for 128-bit security category applications, making them viable replacements in SPHINCS+ 128s and similar PQC signature schemes. Ascon-XOF128’s variable-length output preserves the flexibility of SHAKE while significantly reducing FPGA resource usage. However, its security ceiling of 128-bit preimage resistance limits its applicability in higher security categories (e.g., SPHINCS+ 192s/256s), where SHA3-256 and SHAKE256 offer up to 256-bit resistance.

The combined theoretical and hardware evidence reinforces the case for integrating Ascon-Hash256 and Ascon-XOF128 into post-quantum signature schemes. From a security perspective, both match SHA3-256 and SHAKE128 for category 1 PQC applications while maintaining flexibility through variable-length outputs. From a hardware perspective, FPGA results demonstrate that Ascon-based designs can reduce LUT usage by over 85% and BRAM requirements by more than 90% compared to SHAKE256-based SPHINCS+ while keeping energy consumption competitive [45,62]. This balance of security and implementation efficiency positions Ascon as a strong candidate for low-power, resource-constrained PQC deployments, provided that its use is targeted at security levels where its 128-bit preimage bound remains sufficient.

Empirical SMHasher evaluations further confirm that Ascon hash outputs maintain uniformity and low bias under structured inputs relevant to SPHINCS+ workloads. While not a formal security proof, these results complement the theoretical bounds in Table 6 by demonstrating practical mixing behavior suitable for pseudorandom string derivation and multi-layer Merkle hashing.

### 5.4. Tamper-Evident Fingerprinting and Logging for IoT Devices

In embedded and IoT systems, compact and secure fingerprinting mechanisms are essential for software versioning, device identity, and event stream validation [3]. Traditional cryptographic hashes such as SHA-2 or SHA-3 offer strong security guarantees but often incur prohibitive performance or area costs in constrained environments [66]. Ascon-Hash, with its lightweight sponge construction and constant-time permutation, is particularly suited for these tasks.

To evaluate its suitability for fingerprinting applications, we considered both synthetic and realistic input structures. From the synthetic perspective, the SMHasher suite (Section 5.1) showed that Ascon-Hash256 exhibited a worst-case avalanche bias of only 0.823%, along with minimal structural degradation under Permutation (0.081%) and Cyclic (0.151%) Keyset Tests. These metrics indicate strong diffusion even for low-entropy or repetitive inputs, which is essential for fingerprinting firmware binaries, configuration states, or sensor logs.

From a practical perspective, we simulated a log fingerprinting scenario in Python to illustrate how Ascon-Hash behaves under realistic structured data changes. Ten short log entries (Table 7) were created with minor variations in temperature, timestamp, and status fields to emulate sensor reports or audit entries.

We selected ten adjacent log pairs (e.g., L1 vs. L2, L1 vs. L3, etc.) and computed the Hamming distance between their 256-bit hash outputs [38]. For non-cryptographic hashes (MurmurHash3 and xxHash), which naturally output 128 bits, a “fair” 256-bit construction was used:

$$H_{256}\left(m\right)=H_{128}\left(m\;\|\;0\right)\;∥\;H_{128}\left(m\;\|\;1\right)$$

This avoids the avalanche underestimation caused by zero-padding, which fixes half the bits and artificially reduces the observed diffusion.

For each pair of adjacent log entries (Table 7), we computed the Hamming distance between their 256-bit hash outputs. We evaluated Ascon-Hash256, SHA3-256, SHAKE256 (32-byte output), BLAKE2s-256, and two non-cryptographic hashes (MurmurHash3 and xxHash) lifted to 256 bits via the domain-separated construction.


def bit_diff(h1: bytes, h2: bytes) -> int:
    assert len(h1) == len(h2), “hash lengths must match”
    return sum((a ^ b).bit_count() for a, b in zip(h1, h2))


A cryptographically sound 256-bit hash should flip roughly half its output bits under small input changes; the reference distribution is Binomial (256,0.5) with a mean of 128 and a standard deviation of ≈8 [22,67]. We report the mean ± standard deviation (and 95% confidence intervals where noted) over the ten structured log pairs.

The results in Table 8 show the mean and standard deviation of bit flips across all ten pairs for each algorithm, alongside the Binomial (256,0.5) expectation of the mean ≈128 bits and σ≈8. All cryptographic hashes and the fair 256-bit non-cryptographic hashes produced means within ±3 bits of the theoretical expectation, with Ascon-Hash-256 matching it exactly. This real-data test visually and statistically confirms the strong avalanche property of Ascon-Hash-256 in practical scenarios. For example, changing Temp:22C to Temp:23C (a single character) resulted in 124–139 bit flips out of 256 in our tests, consistent with the SMHasher-reported low avalanche bias (0.823%). Such robustness ensures that even minor field changes in logs yield unpredictable and uniformly distributed digests.

In addition to fingerprinting, Ascon-Hash supports hash chaining for tamper-evident logs [68]:

$$H_{i}=\mathrm{AsconHash}\left(M_{i}\;∥\;H_{i-1}\right)$$

where $M_{i}$ denotes the *i*-th log entry and $H_{i}$ is the corresponding hash chain value (with $H_{0}$ initialized to a fixed constant or system seed). This structure prevents reordering, insertion, or deletion attacks without requiring digital signatures. Because Ascon-Hash outputs exhibit uniform bit distribution (Zeroes Test: 0.322% in SMHasher), chained digests retain high entropy, minimizing bias in audit trails. Such constructions are well suited for offline-capable devices, distributed sensor networks, and ledger-based storage systems, and can naturally extend to Merkle tree commitments for secure data aggregation [69,70].

**Figure 8:**
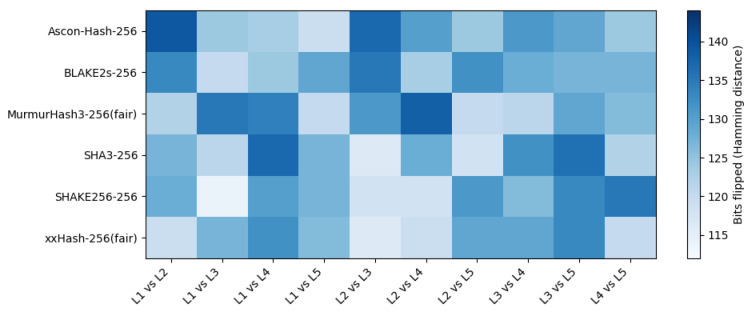
Per-pair avalanche heatmap (bits flipped) for 256-bit outputs across algorithms and adjacent log pairs (Table 7). Non-cryptographic hashes use the fair 256-bit construction $H_{128}\left(m\;\|\;0\right)\;∥\;H_{128}\left(m\;\|\;1\right)$.

Figure 8 visualizes the avalanche behavior per adjacent log pair: rows are hash algorithms and columns are the pairs from Table 7. Each cell encodes the Hamming distance (bits flipped) between the two 256-bit digests; the single-hue blue scale is fixed to 112–144 to emphasize variation around the ideal 128 bits (Binomial (256,0.5)). All algorithms including the fair 256-bit constructions of MurmurHash3 and xxHash exhibit mid-to-high intensities across pairs, with values largely in the 120–140 range. Ascon-Hash-256 aligns with the cryptographic baselines (e.g., high diffusion on L1 vs. L2 and consistently strong values elsewhere), and there are no persistent low-intensity bands across any row or column, indicating no systematic weakness to particular structured changes. The heatmap thus corroborates the aggregate means in Table 8 and supports the claim that Ascon Hash variants deliver near-ideal diffusion on realistic log data.

In summary, the combination of SMHasher-derived statistical strength and the real-world log avalanche test demonstrates that Ascon hash variants deliver cryptographic-grade diffusion at lightweight implementation cost. This makes it a compelling choice for secure fingerprinting, tamper-evident logging, and integrity verification in resource-constrained IoT deployments.

### 5.5. Merkle Tree Diffusion Analysis for Blockchain Integrity in IoT Systems

Merkle trees are a fundamental structure in secure logging [58], firmware authentication, and blockchain systems, enabling tamper-evident aggregation of records. At their core lies a hash function that recursively compresses variable-length messages into fixed-size digests. To be effective in such a role, a hash function must offer strong collision resistance, uniform diffusion, output unpredictability, and computational efficiency.

Merkle tree-based aggregation refers to the process of recursively hashing individual data elements into a binary tree structure to produce a single root hash that represents the collective integrity of all inputs [71]. This structure supports efficient batch verification, tamper evidence, and inclusion proofs and is foundational in blockchain systems, post-quantum signatures, and authenticated logging.

Ascon hash variants leverage a sponge-based architecture with a compact 320-bit internal permutation, enabling flexible variable-length handling with minimal overhead. This lightweight, reusable design, endorsed and standardized by the NIST [6], makes them particularly well suited for Merkle tree aggregation on resource-constrained platforms such as IoT nodes, edge devices, and FPGAs.

**Figure 9:**
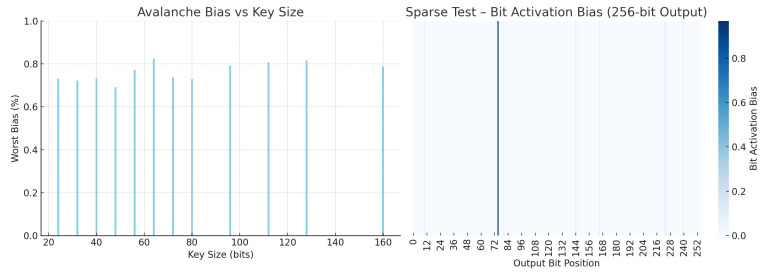
SMHasher analysis of Ascon-Hash. (**Left**) Avalanche bias across varying key sizes shows consistent diffusion (<0.83%). (**Right**) Sparse Test heatmap showing uniform bit activation in the 256-bit output.

We evaluated Ascon-Hash using SMHasher [19] to assess its diffusion behavior and structural robustness (Figure 9). Avalanche testing confirmed that flipping a single input bit results in balanced output changes. Sparse key testing, particularly relevant for structured logs or repetitive data, revealed strong bit dispersion, which enhances collision resistance in real-world log chains.

To evaluate bit-level diffusion within the Merkle tree structure, we implemented a custom Python simulation that perturbs each leaf node individually and computes the resulting bit differences in the root digest. Table 9 summarizes statistical changes in output across different tree levels, highlighting the sensitivity and diffusion strength of Ascon-based hashing.

**Figure 10:**
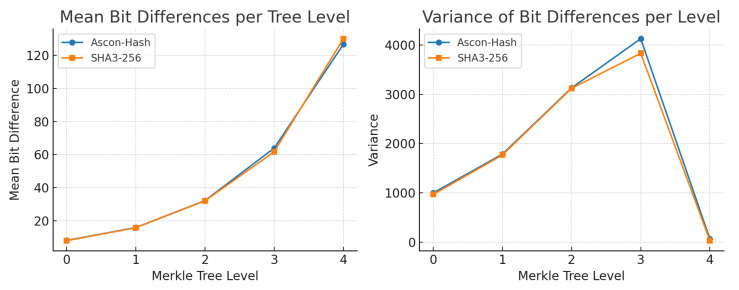
Comparison of Ascon-Hash and SHA3-256 in Merkle trees: (**left**) mean bit differences per tree level; (**right**) variance of bit differences across perturbed inputs. Both functions exhibit strong avalanche effects, with Ascon-Hash showing slightly higher diffusion variance at intermediate levels.

Notably, Ascon-Hash and SHA3-256 show almost identical variance profiles across levels 0–3, indicating similar avalanche strength during internal propagation. At the root level (level 4), SHA3-256 exhibits a marginally higher mean and lower variance, suggesting tighter diffusion consistency at the final aggregation point, as visualized in Figure 10.

The presence of zero-bit differences in levels 0–3 stems from subtree isolation; only nodes along the affected branch are impacted. This effect diminishes at the root level, where all perturbed leaves contribute to observable changes.

**Figure 11:**
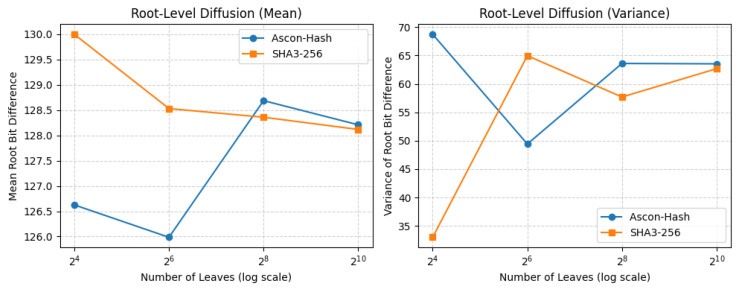
Root-level diffusion behavior as Merkle tree size is scaled from $2^{4}$ to $2^{10}$ leaves for Ascon-Hash and SHA3-256. (**Left**) Mean bit difference in the root digest. (**Right**) Variance of bit differences.

To evaluate how this behavior scales, we extended the analysis across deeper Merkle trees with $2^{4}$, $2^{6}$, $2^{8}$, and $2^{10}$ leaves. For each tree size, we flipped one leaf input and measured the bit difference in the root hash, as shown in Figure 11.

As shown in Table 10, both Ascon-Hash and SHA3-256 converge toward similar mean diffusion values as the tree depth increases, though Ascon exhibits slightly higher variance for small and medium tree sizes.

Our Python-based simulation revealed several critical findings. First, both Ascon-Hash and SHA3-256 exhibit a strong avalanche effect across varying tree sizes. Specifically, a single-bit perturbation in a leaf node consistently produces a root-level bit difference close to 128 out of 256 bits, precisely the expected value for an ideal cryptographic hash function with uniform diffusion. This behavior confirms the robustness of Ascon-Hash in propagating changes throughout the tree structure.

Second, the variance of root-level bit differences stabilizes as tree depth increases. This indicates that deeper Merkle trees exhibit more consistent and predictable behavior in their root digests. Such consistency is essential for applications like blockchain integrity or tamper-evident logs, where deterministic hash outcomes are required for reliable verification and inclusion proofs.

Finally, across all evaluated tree sizes, Ascon-Hash closely mirrors the performance of SHA3-256 in terms of both the mean and variance of diffusion. This suggests that Ascon-Hash can serve as a competitive alternative for secure Merkle aggregation, particularly in constrained-resource platforms such as IoT devices or FPGAs, where computational efficiency and a small hardware footprint are critical.

These findings carry significant implications for real-world deployments. In IoT sensor networks, where Merkle trees typically span 64 to 256 leaves, Ascon-Hash delivers cryptographically secure root digests while maintaining minimal computational overhead. This makes it an ideal candidate for lightweight device authentication [6] and secure data aggregation [71].

For applications in blockchain systems or post-quantum digital signature schemes, such as SPHINCS+, where trees with 512 to 1024 leaves are common, Ascon-Hash continues to maintain uniform and predictable diffusion [62]. This ensures that even large-scale Merkle constructions can benefit from Ascon-Hash’s security properties without sacrificing performance, making it a strong fit for tamper-evident storage, ledger integrity, and quantum-resistant authentication protocols.

Overall, Ascon-Hash matches the diffusion performance of SHA3-256 while offering superior efficiency for hardware implementations, reinforcing its role as a viable and scalable solution for Merkle tree–based applications in constrained environments [72].

## 6. Discussion

This study presents a multi-faceted empirical evaluation of the Ascon hash family, with an emphasis on structural robustness, cryptographic diffusion, and practical applicability in constrained environments. The results demonstrate that Ascon-Hash and Ascon-XOF consistently exhibit low output bias, strong avalanche behavior, and excellent input sensitivity across both synthetic keyset tests and real-world simulations. Compared to SHA3-256, SHAKE256, BLAKE2s, and selected non-cryptographic hashes, Ascon variants show highly competitive performance, particularly in resource-aware deployments where lightweight implementation is critical.

The SMHasher suite analysis in Section 5.1 confirmed that Ascon-Hash delivers excellent bit diffusion, especially under sparse, cyclic, and permutation keyset conditions. These tests modeled common structural patterns in embedded systems, hash tables, and packet headers. Ascon-Hash achieved the lowest Permutation Test bias (0.081%) and top-tier performance in sparse input conditions, outperforming even SHA3-256 in some metrics [19]. These findings validate its robustness against low-entropy or adversarial input structures, making it a strong candidate for secure indexing and fingerprinting tasks.

In Section 5.2, we extended our evaluation to Bloom filter-based replay prevention under realistic nonce indexing scenarios [16]. The Python simulation showed that Ascon-XOF128 consistently maintained the lowest FPR over 200,000 nonce insertions, outperforming SHA3-256, SHAKE256, and MurmurHash3. This result underscores the importance of internal diffusion properties, particularly for the short fixed-length inputs common in edge devices [22]. The sponge structure of Ascon-XOF proved especially effective in distributing entropy uniformly, reducing the likelihood of hash collisions within the Bloom filter [38]. Ascon-XOF’s suitability for lightweight, stateless replay detection was thus empirically validated, highlighting its relevance in CoAP, MQTT-SN, and secure telemetry pipelines [16].

Section 5.3 explored Ascon’s integration into post-quantum signature schemes, specifically referencing the Ascon-Sign proposal [62]. Compared to SPHINCS+-128s and SPHINCS-256, Ascon-Sign demonstrated significantly lower hardware resource requirements and acceptable signing energy costs, despite slightly higher latency. These findings are critical for secure IoT platforms where both memory and power are constrained. The reuse of Ascon-XOF for pseudorandom generation and tree hashing further simplifies hardware implementation and enables streaming-friendly digest computation. This affirms that Ascon variants can serve as cryptographic drop-in replacements in PQC schemes without compromising structural security.

As discussed in Section 5.4, Ascon-Hash proved highly effective for structured fingerprinting scenarios. Simulations of log entries with minimal field changes revealed consistent avalanche scores near 127 bits, on par with SHA3-256 and BLAKE2s. Unlike MurmurHash3, which suffers from poor avalanche behavior, Ascon-Hash maintains high sensitivity across input pairs, ensuring that minor changes in logs or configuration files yield distinct fingerprints [57]. Furthermore, the support for chained hashing enables forward-secure log construction without the need for full Merkle materialization [69]. This makes Ascon-Hash a strong candidate for secure audit trails, clone detection, and software version tracking in embedded devices.

Section 5.5 presented a detailed comparison of Ascon-Hash and SHA3-256 within Merkle tree constructions. Root-level diffusion analysis, conducted using a Python simulation, revealed that both hash functions maintain near-ideal avalanche behavior across tree depths from $2^{4}$ to $2^{10}$ leaves. While SHA3-256 showed slightly tighter variance at the root level, Ascon-Hash exhibited similar mean diffusion and stable variance trends, indicating reliable propagation of leaf-level changes through the aggregation hierarchy. These results, aligned with SMHasher tests, reinforce Ascon-Hash’s viability in Merkle-based systems such as blockchain ledgers, firmware authentication, and post-quantum signature trees [20,42].

Taken together, these results underscore the versatility and efficiency of Ascon hash functions across a range of structural, cryptographic, and embedded contexts. Their ability to simultaneously deliver low overhead and strong diffusion while maintaining hardware friendliness positions them as valuable tools for modern lightweight systems. Future research may focus on extending support in cryptographic libraries, developing streaming interfaces, and verifying side channel resistance in hardware deployments.

While the empirical results demonstrate that Ascon’s hash variants are strong candidates for lightweight, cryptographically secure applications, several practical limitations should be acknowledged before advocating widespread adoption.

First, the Ascon hash variants are relatively new [6] and have not yet achieved widespread integration into major cryptographic libraries such as OpenSSL, Libsodium, or BoringSSL. This limited ecosystem support hinders seamless adoption in production environments that depend on mature, standardized APIs, and may require developers to rely on standalone implementations or custom wrappers.

Second, although the Ascon permutation has undergone public scrutiny as part of the NIST-LWC competition, the hash-specific variants have not yet received the same level of cryptanalytic attention as established hash functions like SHA3-256 or BLAKE2s. While no structural weaknesses are currently known, caution is advised when deploying Ascon hashes in long-term or high-security infrastructure until further independent analysis is available.

Third, while Ascon-XOF and Ascon-CXOF do support variable-length outputs similar to SHAKE256, all Ascon hash variants use fixed-round permutations and fixed-capacity-rate configurations. This design choice simplifies implementation and reduces attack surfaces but limits flexibility in tuning throughput or security margins for domain-specific performance goals. In contrast, SHAKE256 offers more configurability, allowing users to balance output length, digest size, and performance according to application needs [11].

Fourth, due to the sponge-based architecture [38], Ascon hashes require careful handling of input padding, domain separation, and context management to prevent unintended collisions or output reuse in composite constructions such as key derivation functions (KDFs), message authentication codes (MACs), or tree-based hashing. These concerns are not unique to Ascon, but must be explicitly addressed to ensure secure use in multi-stage cryptographic protocols.

Finally, compatibility with existing infrastructure presents a deployment hurdle. Many protocol stacks, digital signature schemes, and secure bootloaders are tightly coupled with SHA2 or SHA3-based primitives [73,74]. Substituting Ascon may require dual-hash compatibility, transitional wrappers, or formal adoption through standards bodies such as the ISO or IETF. Until these integrations mature, the use of Ascon hash functions may be best suited for emerging systems, constrained environments, or research-driven deployments where a low overhead and lightweight properties are prioritized.

Despite these limitations, the Ascon hash family presents a compelling combination of efficiency, structural robustness, and cryptographic soundness. Its inclusion in the NIST-LWC Standard [6] provides a strong foundation for further adoption and analysis, particularly in the growing landscape of secure IoT and embedded applications.

## 7. Conclusions

This study provides a comprehensive empirical evaluation of the Ascon hash family, highlighting its strong diffusion characteristics, structural robustness, and practical applicability across a range of lightweight security scenarios. Through SMHasher benchmarks and Python-based simulations, we demonstrated that Ascon-Hash and Ascon-XOF offer competitive or superior performance compared to established cryptographic and non-cryptographic hash functions in tasks such as Bloom filter indexing, fingerprinting, Merkle tree aggregation, and post-quantum signature integration. A key limitation is the 128-bit ceiling in preimage resistance, which reflects a deliberate efficiency-oriented design trade-off. While this level remains sufficient for IoT, edge, and most lightweight applications, it may restrict adoption in scenarios requiring higher classical or quantum security margins. Further constraints include ecosystem immaturity, fixed parameter configurations, and limited standardization. Nevertheless, Ascon’s sponge-based architecture and hardware-friendly design make it a compelling candidate for secure and efficient deployment in constrained environments such as IoT nodes, edge devices, and blockchain systems. Overall, Ascon hashes should be viewed as promising yet still emerging candidates for lightweight security, with their long-term potential being contingent on sustained cryptanalytic validation and broader ecosystem integration.

## Figures and Tables

**Table 1:** Round constants $C_{i}$ for Ascon permutation rounds [6].

*i*	${\mathrm{const}}_{i}$	*i*	${\mathrm{const}}_{i}$
0	0x000000000000003c	8	0x00000000000000b4
1	0x000000000000002d	9	0x00000000000000a5
2	0x000000000000001e	10	0x0000000000000096
3	0x000000000000000f	11	0x0000000000000087
4	0x00000000000000f0	12	0x0000000000000078
5	0x00000000000000e1	13	0x0000000000000069
6	0x00000000000000d2	14	0x000000000000005a
7	0x00000000000000c3	15	0x000000000000004b

**Table 2:** Lookup table for Ascon’s 5-bit S-box.

*x*	0	1	2	3	4	5	6	7	8	9	a	b	c	d	e	f
SBox(*x*)	4	b	1f	14	1a	15	9	2	1b	5	8	12	1d	3	6	1c
*x*	10	11	12	13	14	15	16	17	18	19	1a	1b	1c	1d	1e	1f
SBox(*x*)	1e	13	7	e	0	d	11	18	10	c	1	19	16	a	f	17

**Table 3:** Ascon Hash variants and their potential applications.

Variant	Output Type	Potential Applications
Ascon-Hash256	Fixed (256 bit)	Message authentication, digital signatures
Ascon-XOF128	Extendable	Key derivation, Merkle trees, PRFs
Ascon-CXOF128	Extendable w/context	Domain-separated hashing

**Table 4:** Consolidated SMHasher results: avalanche, keyset, and bit distribution metrics.

Algorithm	Avalanche Bias (%)	Sparse (%)	Perm. (%)	Cyclic (%)	Zeroes (%)
Ascon-Hash256	0.823	0.968	0.081	0.151	0.322
SHA3-256	1.013	0.581	0.093	0.182	0.330
BLAKE2S-256	0.855	0.594	0.099	0.204	0.424
MurmurHash3	0.787	0.594	0.088	0.183	0.243
xxHash	0.780	0.649	0.084	0.134	0.332

**Table 5:** Comparison of hash-based PQC signature schemes on FPGAs.

Scheme	Device	Sig. Size	LUT	FF	BRAM	DSP	Fclk (MHz)	Power (W)	$E_{\mathrm{sign}}$ (mWs)
Ascon-Sign-128s [63]	Artix-7	7.8 kB	7.0k	5.9k	1.0	0	100	0.208	170
SPHINCS+-128s [64]	Artix-7	8.1 kB	48k	73k	11.5	0	500	9.71	120
SPHINCS+-256s [64]	Artix-7	29.8 kB	51k	75k	22.5	1	500	9.80	188
SPHINCS-256 [65]	Kintex-7	41 kB	19k	38k	36	3	525	4.97	7.6

**Table 6:** Security strengths of Ascon hash variants and SHA3 functions.

Function	Output Size (Bits)	Collision	Preimage	Second Preimage
Ascon-Hash256	256	128	128	128
Ascon-XOF128	*L*	min(L/2,128)	min(L,128)	min(L,128)
Ascon-CXOF128	*L*	min(L/2,128)	min(L,128)	min(L,128)
SHA3-224	224	112	224	224
SHA3-256	256	128	256	256
SHA3-384	384	192	384	384
SHA3-512	512	256	512	512
SHAKE128	*L*	min(L/2,128)	min(L,128)	min(L,128)
SHAKE256	*L*	min(L/2,256)	min(L,256)	min(L,256)

**Table 7:** Structured log entries used in fingerprinting evaluation.

Log ID	Log Entry
L1	Temp:22C;Time:123456
L2	Temp:23C;Time:123456
L3	Temp:22C;Time:123457
L4	Temp:22C;Time:123456;Status:OK
L5	Temp:22C;Time:123456;Status:FAIL
L6	Temp:23C;Time:123457
L7	Temp:23C;Time:123456;Status:OK
L8	Temp:23C;Time:123456;Status:FAIL
L9	Temp:22C;Time:123457;Status:OK
L10	Temp:22C;Time:123457;Status:FAIL

**Table 8:** Mean Hamming distances for structured log pairs (256-bit outputs). Non-cryptographic hashes were evaluated using a fair 256-bit construction: $H_{128}\left(m\;\|\;0\right)\;∥\;H_{128}\left(m\;\|\;1\right)$. Binomial (256,0.5) expectation: mean = 128, σ≈8.

Algorithm	Mean Bits Flipped	Std. Dev.
Ascon-Hash256	128.0	6.08
BLAKE2s-256	127.8	4.45
MurmurHash3-256 (fair)	127.6	6.41
SHA3-256	126.4	6.83
SHAKE256	126.0	6.69
xxHash-256 (fair)	125.0	5.73

**Table 9:** Bit diffusion statistics per Merkle tree level (Ascon-Hash vs. SHA3-256).

Level	Count	Ascon-Hash					SHA3-256				
		**Mean**	**Var**	**Min**	**Max**	**Std. Dev.**	**Mean**	**Var**	**Min**	**Max**	**Std. Dev.**
0	256	8.15	999.55	0	151	31.62	8.02	969.05	0	143	31.13
1	128	15.95	1785.53	0	141	42.24	15.84	1768.46	0	143	42.03
2	64	32.22	3128.11	0	141	55.91	32.08	3119.29	0	148	55.84
3	32	63.91	4124.27	0	149	64.22	61.69	3829.78	0	135	61.90
4	16	126.62	68.73	103	140	8.29	130.00	33.00	121	146	5.74

**Table 10:** Root-level bit diffusion comparison across tree sizes.

Tree Size	#Leaves	Ascon-Hash		SHA3-256	
		**Mean**	**Variance**	**Mean**	**Variance**
Small	$2^{4}$	126.31	68.69	130.00	33.00
Compact	$2^{6}$	125.95	49.05	128.50	64.83
Medium	$2^{8}$	128.60	63.40	128.29	57.94
Large	$2^{10}$	128.18	63.18	128.10	62.88

## Data Availability

The original contributions presented in this study are included in the article. Further inquiries can be directed to the corresponding authors.

## Abbreviations

The following abbreviations are used in this manuscript:

IoT	Internet of Things
AEAD	Authenticated Encryption with Associated Data
FPGA	Field-Programmable Gate Array
ASIC	Application-Specific Integrated Circuit
LUT	Lookup Table
BRAM	Block Random-Access Memory
XOF	eXtendable Output Function
CAESAR	Competition for Authenticated Encryption: Security, Applicability, and Robustness
NIST	National Institute of Standards and Technology
LWC	Lightweight Cryptography
SHA	Secure Hash Algorithm
MMO	Matyas–Meyer–Oseas
RSA	Rivest–Shamir–Adleman
AES	Advanced Encryption Standard
FPR	False-Positive Rate
PQC	Post-Quantum Cryptography
FORS	Forest of Random Subsets
XMSS	eXtended Merkle Signature Scheme
